# Synaptic cell adhesion molecules contribute to the pathogenesis and progression of fragile X syndrome

**DOI:** 10.3389/fncel.2024.1393536

**Published:** 2024-07-03

**Authors:** Shu-Yuan Bai, De-Yang Zeng, Ming Ouyang, Yan Zeng, Wei Tan, Lang Xu

**Affiliations:** ^1^Geriatric Hospital Affiliated to Wuhan University of Science and Technology, Wuhan, China; ^2^Hubei Provincial Clinical Research Center for Alzheimer's Disease, Wuhan University of Science and Technology, Wuhan, China

**Keywords:** synaptic cell adhesion molecules, fragile X syndrome, dendrite spine, synapse pathogenesis, neural circuits

## Abstract

Fragile X syndrome (FXS) is the most common form of inherited intellectual disability and a monogenic cause of autism spectrum disorders. Deficiencies in the fragile X messenger ribonucleoprotein, encoded by the *FMR1* gene, lead to various anatomical and pathophysiological abnormalities and behavioral deficits, such as spine dysmorphogenesis and learning and memory impairments. Synaptic cell adhesion molecules (CAMs) play crucial roles in synapse formation and neural signal transmission by promoting the formation of new synaptic contacts, accurately organizing presynaptic and postsynaptic protein complexes, and ensuring the accuracy of signal transmission. Recent studies have implicated synaptic CAMs such as the immunoglobulin superfamily, N-cadherin, leucine-rich repeat proteins, and neuroligin-1 in the pathogenesis of FXS and found that they contribute to defects in dendritic spines and synaptic plasticity in FXS animal models. This review systematically summarizes the biological associations between nine representative synaptic CAMs and FMRP, as well as the functional consequences of the interaction, to provide new insights into the mechanisms of abnormal synaptic development in FXS.

## 1 Introduction

The human brain houses over 100 billion neurons, which are interconnected through trillions of synapses to form a vast neural network. Synapses serve as crucial nodes for information transmission between neurons, maintaining the integrity of neuron structure and function, and ensuring efficient information transfer within the neural network. A complete synapse consists of three parts: the presynaptic nerve terminal, the synaptic cleft, and the postsynaptic nerve terminal. These components play vital roles in information processing, transmission, and the formation and maintenance of neural circuits during normal brain functioning, making them critical factors that impact behavior (Yogev and Shen, 2014; Rudenko, 2017; Südhof, 2017; Liu, 2019).

**Figure 1:**
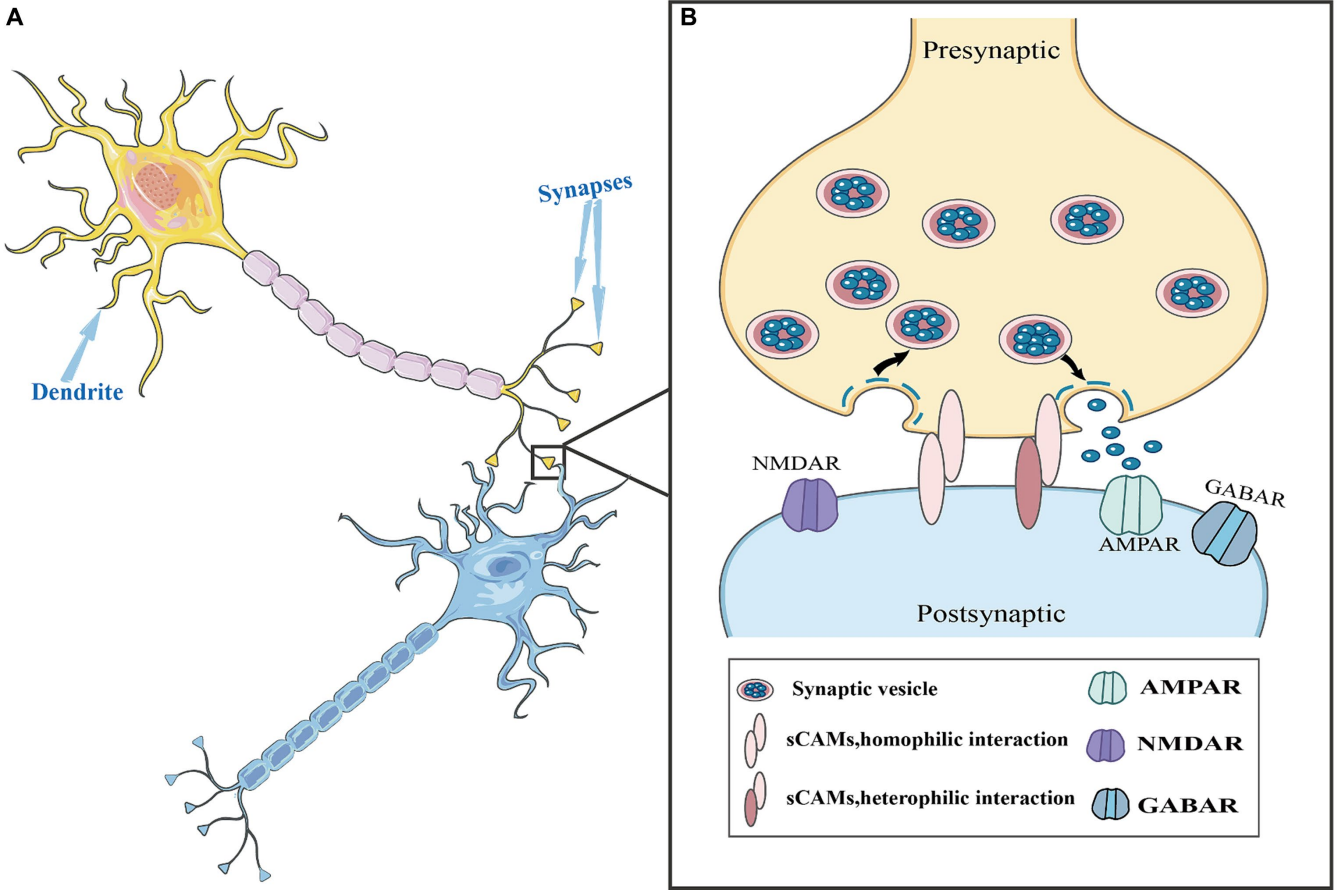
Synaptic cell adhesion molecules (CAMs) in the synaptic structure. **(A)** Neurons are composed of cell bodies and processes. The process is divided into two types: axons and dendrites. Dendrites are generally short and thick, with many branches, and these short branches expand the area of the neuron to receive information. The axon is thin and long, with only one, also known as the nerve fiber. **(B)** A complete synapse consists of three parts: presynaptic nerve terminals, a synaptic cleft, and postsynaptic nerve terminals. During information transmission process, synaptic CAMs establish homophilic or heterophilic interactions between presynaptic and postsynaptic membranes. Additionally, they are involved in the formation and maturation of synaptic vesicles and regulated vesicle release.

**Figure 2:**
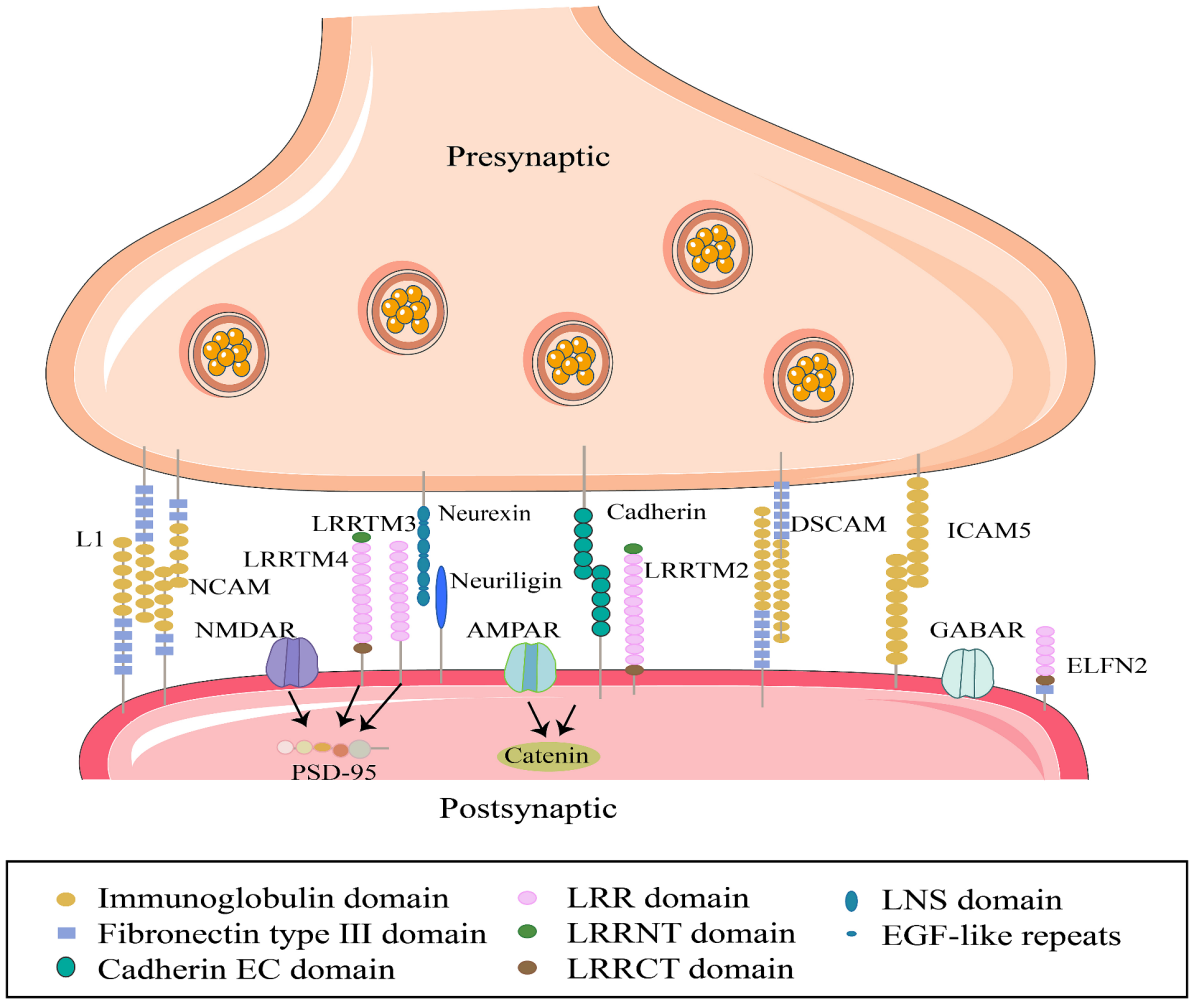
Interactions between specific synaptic cell adhesion molecules in synaptic membranes. Members of the SCAM family, from left to right: L1CAM, L1 cell adhesion molecule; NCAM, Neural cell adhesion molecule; LRRTM 4, Leucine-rich repeat sequences and transmembrane domains; LRRTM 3; Neurexin (neuronal protein); neuroligin (neural glycoprotein); cadherins (calcium-binding proteins); LRRTM 2; DSCAM; ICAM5, Intercellular adhesion molecule; ELFN2, Extracellular leucine-rich repeat sequences and fibronectin type III domain, express synaptic localization functions, participate in synapse generation and maintenance by interacting with cytoskeletal proteins and receptors on cell membranes.

Synaptic cell adhesion molecules (SCAMs) are a class of membrane surface glycoproteins anchored across synapses that facilitate synaptic development (Shapiro et al., 2007; Rudenko, 2017), which can establish homophilic or heterophilic interactions between presynaptic and postsynaptic membranes (Yamada and Nelson, 2007). These interactions ensure the precise transmission of chemical signals that underpin stable neurobiological structure and function (Figure 1). Synaptic CAMs are crucial for dendritic spine formation, maturation, pruning, differentiation, regulation of synaptic plasticity, and learning and memory (Bukalo and Dityatev, 2012; Südhof, 2018; Taylor et al., 2020; Connor and Siddiqui, 2023; Nabavi and Hiesinger, 2023). They belong to several protein families including immunoglobulin (Ig) superfamily proteins, cadherins, neuroligins, neurexins, and leucine-rich repeat proteins. Numerous studies have identified that various synaptic CAMs are involved in neural activities (Taylor et al., 2020; Figure 2; Table 1).

**Table 1:** Characterization of representative synaptic adhesion molecules and their association with FXS.

SCAM category	Molecular characteristics	Interactions between neurons	FMRP target genes	References
ICAM5	Nine extracellular Ig domains	Homophilic interaction	Yes	Tian et al. (2000); Darnell et al. (2011); Yang (2012); Pei et al. (2020)
L1-CAM	Six Ig domains and five FN III-type domains	Homophilic and heterophilic interactions between neurons.	No source	Kadmon et al. (1990a); Sytnyk et al. (2017); Stoyanova and Lutz (2022)
DSCAM	Ten Ig domains and six FN III-type domains	Homophilic and heterophilic interactions between neurons.	Yes	Darnell et al. (2011); Cvetkovska et al. (2013); Guo et al. (2021); Hizawa et al. (2023)
NCAM	Five Ig domains and two FN III-type domains	Homophilic and heterophilic interactions between neurons.	Yes	Sytnyk et al. (2002); Darnell et al. (2011); Chu et al. (2018); Duncan et al. (2021b)
CDH2	A hydrophobic transmembrane region, extracellular region, and highly conserved intracellular C-terminus.	Homophilic and heterophilic interactions between neurons.	Yes	Obst-Pernberg and Redies (1999); La Fata et al. (2014)
PCDH10	Six extracellular cadherin repeats in the ectodomain, a transmembrane domain, and a unique cytoplasmic domain.	Homophilic interaction	Yes	Hirano et al. (1999); Darnell et al. (2011); Kim et al. (2011); Zhen et al. (2023)
CLSTN1	Two cadherin repeat sequences and a laminin-alpha/neurexin/sex hormone-binding globulin (LNS) domain.	Homophilic interaction	Yes	Darnell et al. (2011); Um et al. (2014)
NRXNs	Neurexins are expressed in the presynaptic membranes of neurons; through interactions with NLGNs, they play crucial roles in the formation and maturation of synapses.	Binding with NLGNs to form an XRXN-NLGN protein network.	XRXN1 and XRXN3 are FMRP target genes.	Darnell et al. (2011); Lai et al. (2016); Südhof (2018); Chmielewska et al. (2019)
NLGNs	NLGN1 and NLGN3 localize to glutamate postsynaptic sites; NLGN2 localizes primarily to GABA synapses.	Binding with XRXNs to form an XRXN-NLGN protein network.	NLGN1, NLGN2, and NLGN3 are FMRP target genes.	Graf et al. (2004); Budreck and Scheiffele (2007); Chubykin et al. (2007); Darnell et al. (2011); Lai et al. (2016); Südhof (2018)
LRRTMs	All four members of the LRRTM family are expressed predominantly in the hippocampus.	No source	No direct biological association	de Wit et al. (2009); Linhoff et al. (2009); Ko et al. (2011)

sCAMs, Synaptic cell adhesion molecules; FMRP, Fragile X messenger ribonucleoprotein; ICAM5, Intercellular adhesion molecule 5; Ig, Immunoglobulin; L1-CAM, l1 neural adhesion molecule; FN III, Fibronectin type III; DSCAM, Down syndrome cell adhesion molecule; NCAM, Neural cell adhesion molecules; CDH2, Neural(n)-cadherin; PCDH10, Protocadherin-10; CLSTN1, Calsyntenin 1; NRXNs, Neurexins; XRXN1, Neurexin-1; XRXN3, Neurexin-3; NLGNs, Neuroligins; NLGN1, Neuroligin-1; NLGN2, Neuroligin-2; NLGN3, Neuroligin-3; and LRRTMs, Leucine-rich repeat transmembrane proteins.

Recently, several synaptic CAMs, such as intercellular adhesion molecule 5 (ICAM5) (Pei et al., 2020), neuroligin-1 (Dahlhaus and El-Husseini, 2010; Lai et al., 2016), N-cadherin (La Fata et al., 2014), L1-CAM (Djabali et al., 1990), and calsyntenin 1 (CLSTN1) (Cheng et al., 2019), have been found to aberrantly intervene in the pathological phenotype of fragile X syndrome (FXS), providing insights into the pathogenic mechanisms underlying FXS. As the most common intellectual developmental disorder (Jacquemont et al., 2007; Hagerman and Hagerman, 2021) and the most common single-gene factor in autism spectrum disorder (ASD) (Bagni et al., 2012; Deng and Klyachko, 2021; Chen Y. S. et al., 2022). FXS is often used to study neurodevelopmental disease mechanisms. A thorough investigation of the association between synaptic CAMs and FXS will provide new insights into abnormal synaptic development in FXS and the value of synaptic CAMs as drug targets in neurodevelopmental diseases. This article reviews the biological associations and functional consequences of nine representative synaptic CAMs in the context of FXS, offers new perspectives for understanding the mechanisms of abnormal synaptic development in FXS, and discusses the value of synaptic CAMs as potential drug targets in neurodevelopmental diseases.

## 2 Fragile X syndrome

**Figure 3:**
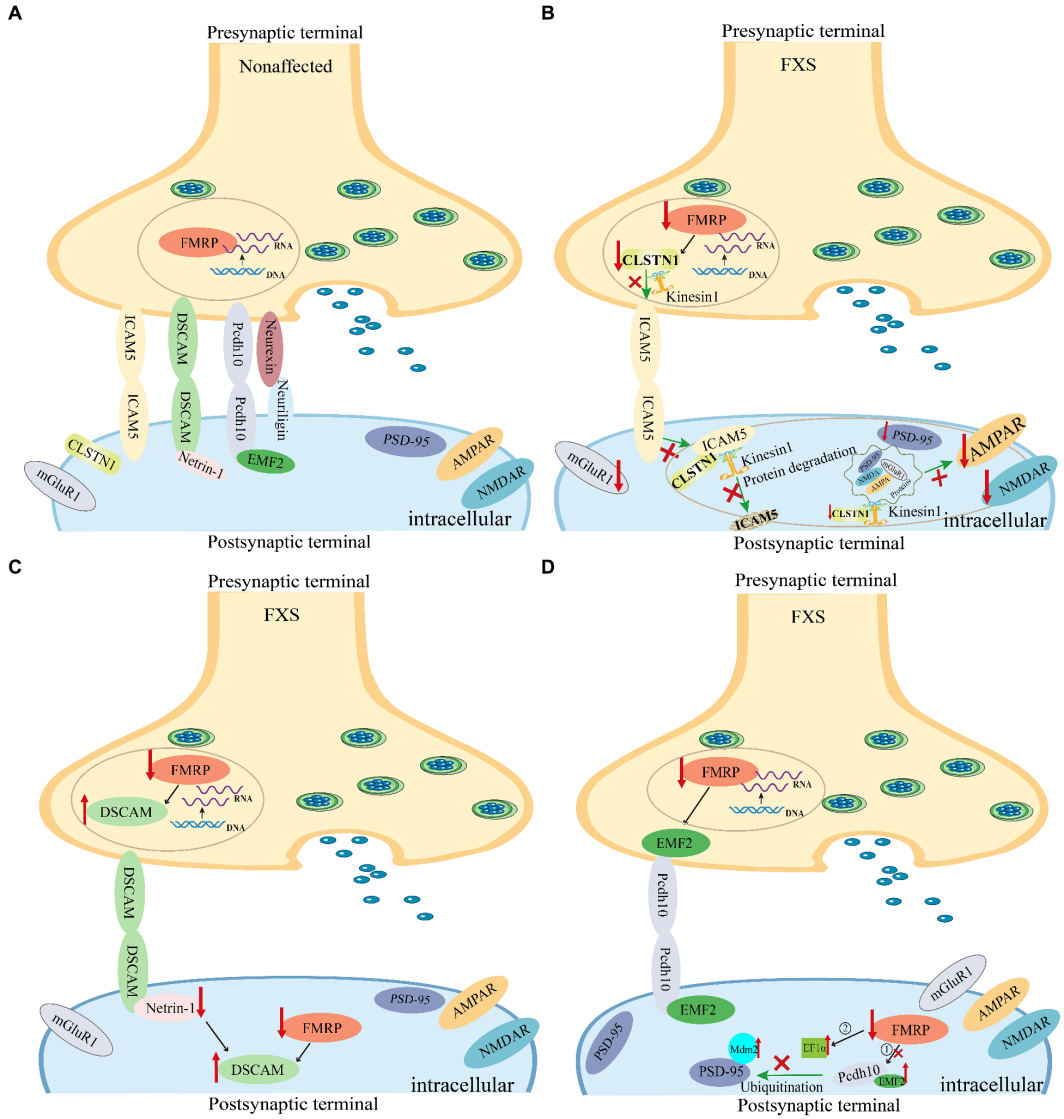
Synaptic cell adhesion molecules are implicated in the abnormal dendritic spine development in fragile X syndrome (FXS) mice. The loss of FMRP in *Fmr1* KO mice leads to abnormal expression of synaptic cell adhesion molecules, thereby causing synaptic dysfunction in FXS. **(A)** The expression of representative synaptic cell adhesion molecules on synaptic membranes under normal conditions. **(B)** Changes in ICAM5 expression in the brains of FXS mice. Both CLSTN1 and ICAM5 are target proteins regulated by FMRP. In various brain regions of the FXS *Fmr1* KO mouse model, reduced expression of CLSTN1 leads to impaired degradation of ICAM5, resulting in the accumulation of ICAM5 on cell membranes, which hinders the development and maturation of dendritic spines and synapses (Cheng et al., 2019; Pei et al., 2020). **(C)** Changes in DSCAM levels in the brains of FXS mice. FMRP binds to and inhibits DSCAM expression. FMRP plays a role in the translational regulation of DSCAM mRNA in hippocampal dendritic spines. Further, DSCAM mRNA is localized to the dendritic terminals of mouse hippocampal neurons and is dynamically regulated by the axon-guidance molecule netrin-1 (Jain and Welshhans, 2016). **(D)** Transcription factors MEF2 and FMRP cooperatively regulate the expression of protocadherin-10 (PCDH10) (Tsai et al., 2012). Nuclear MEF2 activation initiates Mdm2 transcription, resulting in PSD-95 ubiquitination. Pcdh10 binds to ubiquitinated PSD-95 for proteasome degradation, resulting in synapse elimination. In the absence of FMRP, basal levels of MDM2 phosphorylation are elevated, and MEF2 activation fails to cause dephosphorylation of MDM2. Additionally, increased EF1α protein levels prevent MDM2 ubiquitination of PSD-95 after MEF2 activation, thereby blocking PSD-95 degradation and MEF2-induced synapse elimination (Tsai et al., 2017). FMRP, Fragile X messenger ribonucleoprotein; ICAM5, Intercellular adhesion molecule 5; CLSTN1, Calsyntenin 1; DSCAM, Down syndrome cell adhesion molecule; PCDH10, Protocadherin-10; MEF2, Myocyte enhancer factor 2; EF1α, Elongation factor 1α; MDM2, Mouse double minute 2.

Fragile X syndrome affects approximately 1 in 3,600 males and 1 in 4,000–6,000 females (Tassone et al., 2012; Sitzmann et al., 2018; Protic et al., 2022; Elhawary et al., 2023). Approximately 60% of males with FXS meet criteria for ASD (Hagerman and Hagerman, 2021; Marlborough et al., 2021) and have some symptoms of autism such as poor eye contact or repetitive behavior like hand flapping (Roberts et al., 2007; McDuffie et al., 2015), 23% patients with FXS experience seizures (Tondo et al., 2011; Hagerman et al., 2017; Hagerman and Hagerman, 2021). FXS is caused by the abnormal expansion of CGG trinucleotide repeats (>200 CGG) in the first exon of the *FMR1* gene, leading to hypermethylation in the promoter region and silencing of FMR1 protein expression. This, in turn, leads to reduced or absent expression of the target protein, fragile X messenger ribonucleoprotein (FMRP) (Verkerk et al., 1991; Ceolin et al., 2017; Richter and Zhao, 2021). FMRP, a multifunctional RNA-binding protein distributed in neuronal cell bodies, dendrites, and dendritic spines, regulates approximately 4% of brain mRNA stability, intracellular transport, translation, and even post-translational modification in an activity-dependent manner related to neural development and synaptic plasticity (McLennan et al., 2011; Nalavadi et al., 2012; Tassone, 2014; Kurosaki et al., 2022). The core pathological features of FXS include dendritic spine malformations (Berry-Kravis, 2014; Nishiyama, 2019) and synaptic plasticity impairment (Bagni and Zukin, 2019), a profile of neuropathological changes that are shared with bipolar and attention deficit disorders, depression, and schizophrenia (Fernández et al., 2013; Bagni and Zukin, 2019). Consequently, individuals with FXS and FXS animal models exhibit numerous thin and elongated immature dendritic spines in neurons, which contribute to abnormal neuronal connections and FXS-associated behavioral and cognitive impairments (Portera-Cailliau, 2012; Quach et al., 2021). FMRP controls synaptic function by interacting with unique postsynaptic membrane substrates, particularly synaptic CAMs such as neuroligin-1, N-cadherin, L1-CAM, and ICAM5. Therefore, dysregulation of interactions between FMRP and synaptic CAMs is associated with psychiatric and neurological disorders (Figure 3A).

## 3 Associations between synaptic adhesion molecules and FXS

### 3.1 Immunoglobulin superfamily

The characteristic feature of IgSF members is proteins with a highly conserved Ig-like domain that is predominantly observed in cell surface proteins owning to its capacity to resist proteolysis. It has evolved through mutation and selection to serve many biological functions, including growth and development, signaling, adhesion, and protein−carbohydrate interactions. The Ig domain fold, in addition to its closely related fibronectin type III (FnIII) fold, provides an optimal structural foundation for the generation of a vast array of potential protein–protein interaction surfaces (Rougon and Hobert, 2003; Srinivasan and Roeske, 2005; Angata and Varki, 2023). This superfamily includes various subtypes of neuron-specific intercellular adhesion molecules (ICAMs). In vertebrates, ICAMs mediate interactions between nerve cells and within certain IgSF subfamilies by binding to each other through homophilic and heterophilic interactions, thereby forming a small interaction network (Zinn and Özkan, 2017). We addressed the role of several Ig superfamily cell adhesion molecules [i.e., ICAM5, L1 neural adhesion molecule (L1-CAM), Down syndrome cell adhesion molecule (DSCAM), and neural cell adhesion molecule (NCAM)] in FXS.

#### 3.1.1 Intercellular adhesion molecule 5

##### 3.1.1.1 Characteristics and function

Intercellular adhesion molecule 5 is the first dendrite-specific cell adhesion molecule to be identified and the transmembrane protein discovered to form and maintain filamentous dendritic spines, inhibiting dendritic spine maturation. Unlike other ICAM family members, ICAM5 is exclusively expressed in excitatory neurons of the forebrain, also known as telencephalin (Tian et al., 2007; Gahmberg et al., 2008, 2014; Paetau et al., 2017). ICAM5 possesses nine extracellular Ig domains, making it the largest member of the ICAM subfamily (Yang, 2012). It mediates homophilic binding between neurons (Tian et al., 2000). ICAM5 subcellularly localizes to the postsynaptic neuron soma and dendritic membrane but is not expressed in the axon (Yang, 2012). During the early formation of synapses, the immunoreactivity of ICAM5 gradually increases in filamentous dendritic spines and thin dendritic protrusions, aiding in the formation and maintenance of their filamentous morphology. However, its expression decreases or disappears in mature dendritic spines, promoting spine maturation (Raemaekers et al., 2012; Kelly et al., 2014; Pei et al., 2020). Studies have also shown that ICAM5 loss in neurons increases dendritic spine maturation, while overexpression impedes maturation and increases the dendritic protrusion count (Matsuno et al., 2006; Kelly et al., 2014). Furthermore, research suggests a close correlation between the expression level and functional status of ICAM5 and the transition from dendritic filopodia to mature dendritic spines. This correlation is linked to dendritic spine development, synaptic plasticity, neural circuit formation, and even learning and memory (Yang, 2012; Cheng et al., 2019).

##### 3.1.1.2 Implication of ICAM5 in FXS or FMRP pathologies

Darnell et al. (2011) identified ICAM5 as a FMRP target gene. Research by Zeng et al. in FXS animal models, specifically *Fmr1* gene knockout (KO) mice, validated ICAM5 as an mRNA target of FMRP, highlighting its crucial role in dendritic spine maturation and cognitive dysfunction associated with FXS (Pei et al., 2020). They found an abnormal upregulation of ICAM5 protein expression during critical periods of synaptic development in *Fmr1* KO mice, providing insights into the molecular mechanisms underlying dendritic spine maturation impairments in FXS. Pei et al. also observed increased ICAM5 expression in various brain regions of *Fmr1* KO mice, including the hippocampus, frontal cortex, and amygdala. Furthermore, they revealed that CLSTN1, another target of FMRP, plays a crucial role in mediating the redistribution of ICAM5 in the postsynaptic membrane (Cheng et al., 2019; Pei et al., 2020) (Figure 3B).

Identifying FMRP target mRNAs is crucial for understanding the pathogenesis of FXS. However, obtaining a complete understanding of the specific responses of FMRP and identifying its targets is challenging. Accurately determining FMRP targets remains a major challenge, and each newly identified target represents a significant step in exploring FMRP functions (Fernández et al., 2013), providing a direction for future research.

##### 3.1.1.3 Potential pharmacological targets

Pei et al. (2020) alleviated the behavioral deficits in Fmr1 KO mice through genetic ICAM5 intervention, which may provide therapeutic benefits for the treatment of FXS cognitive impairment and other neurodevelopmental disorders. In the future, this molecule could be used as an important drug target for the treatment of behavioral defects in FXS patients.

Additionally, Tian et al. (2007) validated ICAM5 as a substrate for matrix metalloproteinases-2 (MMP-2) or MMP-9 using various experimental approaches and concluded an important role of MMP-mediated ICAM5 proteolytic cleavage in the regulation of dendritic spine development. Conant et al. (2010) found that N-methyl-D-aspartic acid (NMDA) can stimulate rapid shedding of ICAM5 from cortical neurons in dissociated cell cultures. Such shedding is diminished by the pretreatment of cultures with inhibitors that target MMP-9. MMP-2 and MMP-9 are the most abundantly expressed in the developing brain. MMP-2 is mainly found in astrocytes, while MMP-9 is highly expressed in neuronal cell bodies and dendrites (Ayoub et al., 2005; Tian et al., 2007). The expression and activity of MMP-9 have been shown to depend on NMDA receptor activation and long-term potentiation (Meighan et al., 2006; Nagy et al., 2006; Wright et al., 2007). Growing data also suggest the association of MMP-9 (Meighan et al., 2006; Nagy et al., 2006; Huntley, 2012) with dendritic spine remodeling, synaptic plasticity, learning, and memory formation. A mechanism by which MMPs may rapidly modulate synaptic structure and function is through their ability to cleave specific synaptic cell adhesion molecules (Conant et al., 2010). MMPs cleave ICAM5 in a rapid, neuronal activity-dependent manner. Moreover, MMP-mediated proteolysis is associated with LTP (Conant et al., 2010). To better understand the relationship between ICAM5 and MMP-9, Kelly et al. (2014) also explored ICAM5 expression in MMP-9 null animals. Recent studies have shown that the synaptic translation of MMP-9 is regulated by FMRP (Dziembowska and Wlodarczyk, 2012; Janusz et al., 2013; Gkogkas et al., 2014; Lepeta et al., 2017; Aishworiya et al., 2023). Interestingly, aberrations in dendritic spines that are observed in FXS patients (Rudelli et al., 1985) and *Fmr1* KO mice (Comery et al., 1997) have been linked to elevated synaptic levels of MMP-9 (Dziembowska and Wlodarczyk, 2012; Janusz et al., 2013; Lepeta et al., 2017). Clinical trials have reported that minocycline, a broad-spectrum tetracycline antibiotic (Yau et al., 2018), improves cognition and aberrant social behaviors in FXS subjects (Sidhu et al., 2014). In the *Fmr1* KO mice, an abnormally elevated expression of MMP-9 in the brain was pharmacologically downregulated after treatment with minocycline (Dziembowska et al., 2013), while genetic removal of MMP-9 rescued the symptoms of FXS (Sidhu et al., 2014). These data suggest that targeting MMP-9, even in late development, may reduce FXS symptoms. However, it remains to be explored whether alleviation of FXS symptoms by genetic removal of MMP-9 is associated with MMP9-mediated ICAM5 elimination. The specific association between the molecular mechanisms of MMP-9 and ICAM5 may reveal new avenues for individualized treatment of neurodevelopmental disorders, especially FXS, in the future.

#### 3.1.2 L1 neural adhesion molecule

##### 3.1.2.1 Characteristics and function

L1 neural adhesion molecule is a transmembrane protein encoded by the first X-linked gene identified in the IgSF (Djabali et al., 1990). The L1-CAM protein consists of six Ig-like domains, five FNIII-like repeats, a transmembrane, and an intracellular domain (Crossin and Krushel, 2000; Sytnyk et al., 2017; Stoyanova and Lutz, 2022). L1-CAM is expressed in the central nervous system by subpopulations of neurons and on glial cells in the peripheral nervous system (Rathjen and Schachner, 1984; Seilheimer and Schachner, 1987). L1-CAM is involved in adhesion between neurons, the formation of neural fiber bundles, and the growth of nerve processes (Moos et al., 1988; Wang et al., 2012; Congiu et al., 2022; Loers et al., 2023). It mediates homophilic and heterophilic interactions between neurons (Kadmon et al., 1990a; Sytnyk et al., 2017; Stoyanova and Lutz, 2022) and cooperates with NCAM through a mechanism termed assisted homophilic adhesion (Kadmon et al., 1990b). As evidenced in murine model systems, L1-CAM also facilitates axon repulsion and growth cone collapse in response to secreted class 3 Semaphorins (Sema3) (Mohan et al., 2019b; Duncan et al., 2021b), which prune distinct populations of dendritic spines during development and homeostatic scaling (Mohan et al., 2019a,b; Murphy et al., 2023a). Sema3 dimers bind to heterotrimeric receptors comprising L1-CAM, neuropilins (Npn1/2), and plexins A (PlexA1-4), activating intracellular signaling through PlexA Ras-GAP activity that results in spine pruning (Duncan et al., 2021a; Murphy et al., 2023a). Genetic knockouts of L1-CAM in mice lead to increased density of immature dendritic spines on the apical dendrites of cortical pyramidal neurons (Murphy et al., 2023b).

##### 3.1.2.2 Implication of L1-CAM in FXS or FMRP pathologies

Djabali et al. (1990) first determined that the *L1-CAM* gene is located in a conserved region of the X chromosome and considered this protein a typical X-linked NCAM. Notably, several genes linked to neuromuscular diseases are also located in this region adjacent to the fragile site connected with intellectual disability (FRAXA), suggesting a possible association between neuromuscular diseases and intellectual disability. Using pulse-field gel electrophoresis, they confirmed the physical connection between *L1*, and other genes located on Xq28, such as genes encoding eye pigment and glucose-6-phosphate dehydrogenase (G6PD). These locations are consistent with those of the X-linked neuromuscular disease mapping region of the L1 molecule (Djabali et al., 1990).

According to the findings of Loers et al. (2023), L1 siRNA has an inhibitory effect on the expression of long-chain autism genes neurexin 1 (*NRXN1*) and neuroligin 1 (*NLGN1*) and mitochondrial-encoding genes such as NADH ubiquinone oxidoreductase core subunit 2 (*ND2*). Additionally, Lai et al. (2016) found that FMRP binds to NLGN1 and NLGN3 mRNA, whereas Dahlhaus and El-Husseini (2010) revealed an association between the core symptoms of FXS and the neurexin-neuroligin network. Other studies have shown that myelin basic protein cleaves L1 and promotes neurite outgrowth and neuronal survival (Lutz et al., 2014), confirming an indirect association between L1-CAM and FMRP. However, to date, there have been no studies showing a direct connection between L1-CAM and FXS or FMRP. Future research should focus on the functional role of the *L1* gene in the neurexin-neuroligin network and explore its implications for dendritic spine abnormalities in FXS.

##### 3.1.2.3 Potential pharmacological targets

A review of pertinent studies has revealed that L1-CAM binds Ankyrin B (AnkB) at a conserved cytoplasmic domain motif (FIGQY), an actin-spectrin adaptor encoded by Ankyrin2, a gene with high confidence in relation to ASDs (Bennett and Healy, 2009; Murphy et al., 2023a). Additionally, L1 knock-in mouse mutants harboring a point mutation at the L1 ankyrin binding site demonstrated augmented spine density in the prefrontal cortex (PFC) (Murphy et al., 2023b). These findings indicate that AnkB may play a vital role in regulating the pruning of dendritic spines *in vivo*. Meanwhile, various studies have indicated that patients with ASD or FXS display elevated spine density of pyramidal neurons in PFC, where essential circuits contribute to social behavior and cognition (Martínez-Cerdeño, 2017; Murphy et al., 2023b). Using mouse models deficient in L1 family members, Murphy et al. investigated the role of L1 and its interaction with AnkB in dendritic spine regulation in L1-null mice. They found that deletion of L1 or mutation of the FIGQY Ankyrin binding site in the cytoplasmic domain of L1 increased the density of spines on apical dendrites of pyramidal neurons in the mouse neocortex (Murphy et al., 2023a). Moreover, they rescued cortical neurons with impaired dendritic spine development by re-expression of the 220 kDa AnkB isoform in a new inducible mouse model (Nex1Cre-ERT 2: Ank2flox: RCE) (Murphy et al., 2023a). The findings of L1 and its interaction with AnkB in dendritic spine regulation provides a new research direction for the association between L1-CAM and neurodevelopmental diseases such as ASD and FXS and reveal potential pharmacological targets.

#### 3.1.3 Down syndrome cell adhesion molecule

##### 3.1.3.1 Characteristics and function

Down syndrome cell adhesion molecule (*DSCAM*) genes are emerging risk genes for ASDs (Varghese et al., 2017; Chen P. et al., 2022). DSCAM is a transmembrane protein belonging to the IgSF class and is classified as a homophilic cell adhesion molecule (Yamakawa et al., 1998; Agarwala et al., 2000; Guo et al., 2021). The DSCAM protein is expressed in the developing nervous system, where it intervenes in various stages of neuronal development. Such effects range from functions in early development (generation, migration, and differentiation) to plasticity and the formation of neuronal networks (Pérez-Núñez et al., 2016). DSCAM is characterized by a large extracellular region, comprising 10 Ig and 6 FN III domains. Additionally, the intracellular domain lacks identifiable motifs (Ly et al., 2008). DSCAM can mediate cell adhesion by forming homophilic dimers between cells and plays a crucial role in neural development by participating in several processes such as axon collateral guidance, dendritic branching, and targeted synaptic formation (Garrett et al., 2012; He et al., 2014; Li et al., 2015; Mitsogiannis et al., 2020; Guo et al., 2021). Recent reports have identified neuroligin 1 (NLGN1) as a novel heterophilic partner that interacts with the extracellular domain of DSCAM (Chen P. et al., 2022). DSCAM on Purkinje cell membranes interact in a heterophilic manner with the glutamate transporter GLAST in astrocytes (Hizawa et al., 2023; Dewa et al., 2024). In Drosophila, DSCAM exhibits remarkable genetic diversity, with tens of thousands of splicing isoforms that modulate the specificity of neuronal wiring. Notably, this splice variant diversity of DSCAM is absent in vertebrates (Hizawa et al., 2023).

##### 3.1.3.2 Implications of DSCAM in FXS or FMRP pathologies

A growing body of evidence supports that mutations in the *DSCAM* gene (Brown et al., 2001; Darnell et al., 2011; Varghese et al., 2017; Mitsogiannis et al., 2020) and increased DSCAM protein expression are associated with FXS pathogenic mechanisms (Sterne et al., 2015; Montesinos, 2017). The FMRP protein binds to DSCAM mRNA (Brown et al., 2001) and inhibits translation of the *DSCAM* gene in *Drosophila* and mammalian brain neurons (Darnell et al., 2011). FMRP regulates *DSCAM* isoform splicing in various cell types to achieve diverse functions (Brown et al., 2001). In *Drosophila* fragile X mutants, the absence of FMRP leads to increased levels of DSCAM protein owing to translational inhibition, impairing precise synaptic targeting and neural circuit function (Cvetkovska et al., 2013). Abnormal axon targeting caused the degradation of sensory circuit function to an extent that affects *Drosophila* perception (Cvetkovska et al., 2013). By reducing DSCAM levels in fragile X mutants, scientists observed a reduction in targeting errors and rescued corresponding behavioral responses. Moreover, dysregulation of DSCAM protein expression promotes abnormal dendritic spine development in FXS (Nimchinsky et al., 2001; Cvetkovska et al., 2013). In the mammalian brain, *DSCAM* is a target gene of FMRP (Darnell et al., 2011). Jain and Welshhans (2016) supported this finding and suggested that FMRP plays a role in regulating the translation of DSCAM mRNA during hippocampal synapse development. They also found that DSCAM mRNA localized to the axons of mouse hippocampal neurons and was dynamically regulated by the axon-guidance molecule Netrin-1 (Figure 3C). Two RNA-binding proteins, FMRP and cytoplasmic polyadenylation element-binding protein, colocalize with DSCAM mRNA and regulate its stability and local translation. Taken together, netrin-1 increases DSCAM protein in growing axons, and overexpression of DSCAM delays axonal growth and branching in mouse cortical neurons, suggesting that netrin-1-induced local translation of DSCAM mRNA is an important mechanism of axonal growth regulation and nervous system development, with increased expression of DSCAM protein leading to structural changes associated with synaptic development (Jain and Welshhans, 2016).

Kim et al. (2013) later showed that DSCAM expression levels are critical in the regulation of presynaptic dendritic development; they detected an association between *Drosophila* FMRP (dFMRP) and DSCAM mRNA in larval brain lysates using RNA immunoprecipitation and concluded that dFMRP binds DSCAM mRNA and regulates DSCAM expression to inhibit presynaptic dendritic spine development. Sterne et al. (2015) used genetic and pharmacological methods in neurons overexpressing DSCAM and in a *Drosophila* FXS model to demonstrate the reversal of cell defects caused by imbalanced DSCAM levels in response to Abelson kinase (Abl) inhibition. Abl is a well-established target for treating chronic myeloid leukemia, and multiple Abl inhibitors are approved by the US Food and Drug Administration (FDA) (Speck-Planche et al., 2012). Furthermore, studies indicate the potential for a genetic interaction between DSCAM and Abl in the development of neurites in the brain of Drosophila embryos (Andrews et al., 2008; Yu et al., 2009; Sterne et al., 2015).The investigation showed that DSCAM must interact with Abl to influence presynaptic terminal growth. Besides, the larger presynaptic terminals seen in fruit fly larvae, which produce too much DSCAM, are a result of the DSCAM protein over activating Abl. These findings raise the interesting possibility that targeting Abl might be a viable therapy for brain disorders caused by increased DSCAM expression. Studies have attempted to rescue the developmental defects caused by DSCAM overexpression using Abl inhibitors (Sterne et al., 2015). Sterne et al. (2015) used two Abl kinase inhibitors to treat fruit fly larvae, and found that this reversed the detrimental effects of extra DSCAM on the larvae’s neural circuit. Furthermore, the drugs repaired neural defects in a fruit fly model designed to reproduce FXS symptoms.

Mitsogiannis et al. (2020) found that DSCAM and DSCAM-like 1 (DSCAML1) are highly expressed in neural populations in the embryonic mouse cortex. Moreover, using animal FXS models, researchers have discovered that regulating DSCAM expression can significantly alleviate signs of disease. Other studies have revealed that the spontaneous loss of mouse *DSCAM* alleles can lead to motor coordination disorders and seizures, with behavioral manifestations similar to those of FXS (Laflamme et al., 2019). Increased occurrence of seizures can be caused by the loss of FMRP function (Musumeci et al., 1999; Kim et al., 2013; Hagerman and Hagerman, 2021), further highlighting the functional significance of dysregulated DSCAM expression in neuronal development.

##### 3.1.3.3 Potential pharmacological targets

A study has demonstrated that DSCAM regulates neuronal delamination by exerting local suppression of the RapGEF2-Rap1-N-cadherin cascade at the apical endfeet in the dorsal midbrain. DSCAM is associated with RapGEF2 to inactivate Rap1, whose activity is required for membrane localization of N-cadherin (CDH2). Among them, RapGEF2 (also known as PDZ-GEF1/RA-GEF1), a Rap1-specific guanine nucleotide exchange factor (GEF), was identified as a DSCAM-interacting protein. These findings shed light on the molecular mechanism by which DSCAM regulates a critical step in early neuronal development (Arimura et al., 2020). Together, the previously outlined findings provide possible targets for the treatment of FXS, suggesting that interventions in biological processes related to DSCAM may improve the symptoms of patients with FXS in the future.

#### 3.1.4 Neural cell adhesion molecules

##### 3.1.4.1 Characteristics and function

Neural cell adhesion molecule (NCAM), an IgSF member, has been identified as a protein target of FMRP (Darnell et al., 2011). NCAM consists of five Ig domains and two FN III domains and is associated with various aspects of synaptic development and function (Sytnyk et al., 2002). NCAM plays a crucial role in the development and maintenance of the nervous system through homophilic and heterophilic interactions (Chu et al., 2018). The absence of NCAM leads to abnormal synaptic differentiation, which not only disrupts synaptic development but also affects synaptic plasticity (Kochlamazashvili et al., 2012). NCAM also possesses signal transduction capabilities associated with neural growth responses mediated by neural cadherin (CDH2) and interacts with L1-CAM, playing a critical role in neurons (Wiertz et al., 2011; Colombo and Meldolesi, 2015). NCAM is crucial not only for the development of the nervous system, but also for maintaining high cognitive functions of the adult brain (Stoyanova and Lutz, 2022).

##### 3.1.4.2 Implication of NCAM in FXS or FMRP pathologies

While there is currently no literature confirming a direct association between NCAM and FMRP, identifying interactions between NCAM and other adhesion molecules, as well as their functions in neural system development, may provide new avenues to explore the relationship between NCAM and FMRP.

##### 3.1.4.3 Potential pharmacological targets

In summary, the results not only reveal biological associations between IgSF members and FMRP and their association with FXS but also offer new insights into therapeutic strategies for FXS and FMRP-related disorders. By studying members of this family, we can gain a deeper understanding of the pathogenic mechanisms underlying FXS and those associated with FMRP, laying the foundation for the development of effective treatments and breakthroughs in clinical therapy.

### 3.2 Calcium adhesion proteins in FXS

Cadherins constitute an essential family of transmembrane glycoproteins (Riggins et al., 1992). This family includes classical cadherins (types I and II), protocadherins, desmosomal cadherins, and various cadherin-related molecules. They play diverse roles in neural induction, neural cell migration, axonal growth, and synapse formation and maintenance (Obst-Pernberg and Redies, 1999; Halbleib and Nelson, 2006; Sanes and Zipursky, 2020).

#### 3.2.1 Neural cadherins

##### 3.2.1.1 Characteristics and function

Classical cadherins of the calcium adhesion protein family include E-cadherin (CDH1) and neural cadherin (CDH2). CDH1 is a type I transmembrane glycoprotein located in the adhesive junctions and in the basolateral membrane of epithelial cells. It consists of a large extracellular domain, a transmembrane segment, and a conserved cytoplasmic domain (van Roy and Berx, 2008; Wilkerson et al., 2023). There are relatively rare studies on these proteins related to FXS.

Neural(n)-cadherin, first discovered at synapses, is a calcium-dependent single-pass transmembrane glycoprotein that is mainly expressed on postsynaptic membranes (Obst-Pernberg and Redies, 1999). CDH2 plays a crucial role in both homophilic and heterophilic adhesions at synapses and significantly influences neural system development and functional regulation. Its structure comprises a hydrophobic transmembrane region, an extracellular region, and a highly conserved intracellular C-terminus. The intracellular domain associates with the actin cytoskeleton via p120-catenin, α-catenin, and β-catenin, forming the adherens junction (Angst et al., 2001; Marie et al., 2014; Halperin et al., 2021). Since β-catenin is an effector factor in the canonical Wnt/β-catenin pathway, CDH2 can also modulate signal transduction via Wnt/β-catenin in multiple ways (Marie et al., 2014; Yang et al., 2022). CDH2 is involved in multiple aspects of axon development and morphogenesis, including axon extension, fasciculation, and target selection (Jontes, 2018). It helps establish neuronal polarity in the developing cortex and initiates axon outgrowth (Xu et al., 2015).

##### 3.2.1.2 Implication of CDH in FXS or FMRP pathologies

La Fata et al. (2014) suggested that CDH2 mRNA is a key target of FMRP during early development and that its reduction leads to delayed development of cortical neurons in patients with FXS. CDH2 also plays a crucial role in the multipolar-to-bipolar neuronal transition during brain development. Additionally, studies using diffusion tensor imaging and magnetic resonance imaging have shown abnormal structural connections in the brains of young patients with FXS. In FXS mouse models, researchers found that FMRP regulates the positioning of cortical plate neurons during embryonic development, thereby affecting the multipolar-to-bipolar transition in these neurons. Correcting this abnormality is possible by reintroducing FMRP or CDH2 during embryonic development. Stan et al. (2010) also discovered that CDH2 interacts with the scaffolding molecule S-SCAM to control the accumulation of vesicles during synaptic development by binding to NLGN1. The precise molecular mechanism underlying the association between CDH2 and FXS remains to be elucidated. However, this represents a promising avenue for future investigation.

##### 3.2.1.3 Potential pharmacological targets

A study has indicated that FMRP coordinates Wnt/β-catenin signaling during corticogenesis (Casingal et al., 2020), and CDH2, as one of the targets, simultaneously participates in the Wnt/β-catenin pathway (Yang et al., 2022). In addition, some results suggest that PPARγ agonists such as pioglitazone, rosiglitazone, and the synthetic agonist GW1929, are used as therapeutic agents in neurological disorders. These compounds interact with intracellular transduction signals (e.g., GSK3β, PI3K/Akt, Wnt/β-catenin, Rac1, and MMP-9). It appears that interaction with these pathways may improve memory recognition in FXS animal models (Farshbaf et al., 2014). Taken together, these associations may provide new research directions to explore the role of CDH2 in mediating the Wnt/β-catenin signaling pathway in FXS. The Wnt/β-catenin signaling pathway may be considered a new target for FXS treatment.

#### 3.2.2 Protocadherins

##### 3.2.2.1 Characteristics and function

Protocadherins (PCDHs) are predominantly expressed in the nervous system and constitute the largest subgroup within the calcium adhesion protein superfamily, comprising more than 80 genes, including 60 genes in the *α-*, *β-*, and *γ-PCDH* gene clusters and non-clustered *δ-PCDH* genes (Keeler et al., 2015). PCDHs and other atypical cadherins have been shown to play roles in dendrite development and branching and regulation of dendritic spines. They function through homophilic adhesion between neurons (Hoshino et al., 2023).

##### 3.2.2.2 Implication of PCDH10 in FXS or FMRP pathologies

Here, we focus on PCDH10, also known as OL-protocadherin (Morishita and Yagi, 2007), which contains six extracellular cadherin repeats in the ectodomain, a transmembrane domain, and a unique cytoplasmic domain (Hirano et al., 1999; Kim et al., 2011; Zhen et al., 2023), and is a protein target of FMRP (Darnell et al., 2011). Tsai et al. (2012) reported that PCDH10 is an ASD gene necessary for activity-dependent elimination of excitatory synapses in mice. Myocyte enhancer factor 2 (MEF2) and FMRP collaboratively regulate PCDH10 expression in dendrites. MEF2-induced synapse elimination requires FMRP (Pfeiffer et al., 2010). MEF2 induces PCDH10-dependent degradation of PSD-95 by transferring ubiquitinated PSD-95 to the proteasome. Thus, without MEF2 activation, PSD-95 degradation is not expected to occur. Tsai et al. (2017) discovered that, in FMRP-deficient neurons, increased levels of eukaryotic translation elongation factor 1α (EF1α) prevented mouse double minute 2 (MDM2) ubiquitination of PSD-95 after MEF2 activation, blocking MEF2-induced PSD-95 degradation and synapse elimination (Figure 3D).

##### 3.2.2.3 Potential pharmacological targets

Now that PCDH10 is known to target FMRP and MEF2, respectively, perhaps we can further study the relationship between these three in the mouse model of FXS, which may become a potential target for treating FXS.

#### 3.2.3 Calsyntenin family of atypical cadherins

##### 3.2.3.1 Characteristics and function

The CLSTN family of atypical cadherins (Südhof, 2021; Liu et al., 2022) includes calsyntenin 1 (CLSTN1), calsyntenin 2 (CLSTN2), and calsyntenin 3 (CLSTN3). All three CLSTN proteins are expressed in the postsynaptic membranes of neurons (Vogt et al., 2001; Hintsch et al., 2002; Um et al., 2014). CLSTN1 is a type I transmembrane protein with an extracellular domain containing two cadherin repeat sequences and a laminin-alpha/neurexin/sex hormone-binding globulin (LNS) domain (Um et al., 2014). It plays a crucial role in mediating dendritic spine development, synaptic plasticity, and neural circuit formation (Alther et al., 2016; Cheng et al., 2019).

##### 3.2.3.2 Implication of CLSTN1 in FXS or FMRP pathologies

A recent study suggest that CLSTN1 is an important target of FMRP (Darnell et al., 2011). In animal models of FXS, Cheng et al. (2019) first confirmed interactions between CLSTN1 and ICAM5 in the regulation of dendritic spine maturation, demonstrating a key role for CLSTN1 in the development of dendritic spines in *Fmr1* KO mice. They further revealed that CLSTN1 is a target of FMRP and that CLSTN1 expression was reduced in multiple brain regions in *Fmr1* KO mice, including the cerebellum, resulting in impaired protein transport function. This phenomenon ultimately leads to the accumulation of ICAM5 on cell membranes, further impeding the development and maturation of dendritic spines and synapses and causing abnormal spatial and social learning behavior in *Fmr1* KO mice (Cheng et al., 2019; Pei et al., 2020).

Furthermore, other studies suggest that CLSTN3 can form a synaptic adhesion complex with α-NRXNs to induce presynaptic differentiation in developing neurons, thereby participating in synapse formation, regulating synaptic function, and affecting neuron development (Pettem et al., 2013; Um et al., 2014; Gomez et al., 2021; Liu et al., 2022). Currently, there is no clear evidence of a direct association between CLSTN2, CLSTN3, and FMRP. However, their cooperative interactions with other synaptic adhesion molecules that affect the development of synapses make these molecules worth exploring in FXS.

##### 3.2.3.3 Possible pharmacological targets

Taken together with the link between CLSTN1 and ICAM5 above, we may try to genetically intervene in CLSTN1, which provides us with a new research idea to further explore the potential pharmacological targets of FXS.

### 3.3 Neuroligins and neurexins in FXS

#### 3.3.1 Characteristics and function

Neuroligins (NLGNs) are a small family of postsynaptic transmembrane proteins that induce presynaptic differentiation in both glutamate and GABA axons (Graf et al., 2004; Lai et al., 2016). NLGNs comprise the products of three genes: *NLGN1*, *NLGN2*, and *NLGN3*. NLGN1 and NLGN3 localize to postsynaptic sites of Glutamatergic neurons, whereas NLGN2 localizes primarily to GABA synapses (Graf et al., 2004; Chubykin et al., 2007). NLGN3 forms heterodimers with NLGN1 or NLGN2 and acts at synapses (Budreck and Scheiffele, 2007). Notably, NLGNs are the first ligands for presynaptic adhesion molecules such as neurexins (NRXNs), and they play crucial roles in synapse formation and maturation (Ichtchenko et al., 1995; Scheiffele et al., 2000; Chanda et al., 2017; Varghese et al., 2017; Kasem et al., 2018; Südhof, 2018). NRXNs are encoded by three genes: *NRXN1, NRXN2,* and *NRXN3*. Each gene encodes two forms, α-NRXNs and β-NRXNs, of the single-pass transmembrane proteins (Südhof, 2017). There are thousands of splice variants, allowing NRXNs to perform diverse roles in the formation and function of synapses and ensuring normal nervous system operation (Ullrich et al., 1995; Missler and Südhof, 1998; Tanaka et al., 2011; Lai et al., 2016; Lin et al., 2023).

#### 3.3.2 Possible implication of XRXN–NLGN protein in FXS or FMRP pathologies

Dahlhaus and El-Husseini (2010) first provided evidence for the involvement of the XRXN–NLGN protein network in core FXS symptoms. Other studies have suggested that disrupted signaling in the trans-synaptic pathway involving NLGNs and NRXNs is common in other types of ASD (Trobiani et al., 2020). Chanda et al. (2017) systematically analyzed the impact of conditional genetic deletions of major NLGN isoforms, including NLGN1, NLGN2, and NLGN3, on cultured mouse hippocampal and cortical neurons, and revealed that the absence of NLGNs, either individually or in combination, had no effect on synaptic quantity but selectively impaired excitatory or inhibitory synaptic function in an isoform-specific manner, ultimately leading to a reduction in the distribution of neurotransmitter receptor synapses. Conversely, overexpression of NLGN1 increased synaptic quantity without affecting dendritic spine number. These results indicate that overexpression and RNAi-mediated knockdown of NLGNs lead to a significant increase and decrease, respectively, in synaptic density. Although NLGN genetic deletion has a relatively minor impact on synaptic quantity, it severely impairs synaptic function. Darnell et al. (2011) and Lai et al. (2016) found that FMRP binds to NLGN1 and NLGN3 mRNA. In wild-type mice, sex differences exist in the expression of NLGN2, NRXN1, NRXN2, and NRXN3 mRNA in the hippocampal region and NRXN3 mRNA in the somatosensory cortex region. In contrast, *Fmr1* KO mice exhibited sex differences in the expression of NLGN3, NRXN1, NRXN2, and NRXN3 mRNA in the hippocampal region and NLGN1, NRXN2, and NRXN3 mRNA in the somatosensory cortex region. These findings provide a basis for neuroanatomical mapping of NLGNs and NRXNs during postnatal development in WT and *Fmr1* KO mice. Differences in the expression of these synaptic proteins during development may lead to long-term differences in central nervous circuitry and synaptic function (Lai et al., 2016).

Chmielewska et al. (2019) further confirmed the association between FMRP and NLGN1, NLGN2, and NLGN3 mRNA in synaptic bodies and neuron cultures. Their studies confirmed the synaptic regulation of NLGN1, NLGN2, and NLGN3 mRNA by FMRP during local translation. In an *Fmr1* KO mouse model, increased NLGN levels lead to elevated expression of NLGN1 and NLGN3 in the postsynaptic membrane. Furthermore, they found that NLGN synaptic levels were precisely and dynamically regulated through rapid protein degradation under NMDA stimulation in both wild-type and *Fmr1* KO mice (Chmielewska et al., 2019). Additionally, Budreck et al. (2013) found that NLGN1 controlled the synaptic abundance of NMDA-type glutamate receptors through extracellular coupling. In summary, Chmielewska et al. linked abnormal synaptic expression of NLGNs with FMRP, providing evidence for the molecular basis of FXS.

Furthermore, other research indicate that missense mutations in NRXN1 may be associated with neurodevelopmental disorders beyond ASD and/or schizophrenia (Ishizuka et al., 2020), which involves cytoplasmic *Fmr1* interacting protein 1 (CYFIP1). Bachmann et al. (2019) discovered that functional impairment of the monomeric form of CYFIP1 in *Fmr1* KO mice resulted in changes in dendritic spine morphology and synaptic plasticity. Further investigation revealed a synaptic protein cluster centered around CYFIP1 and NLGN3 (Tanaka et al., 2011). This cluster not only regulates dendritic spine morphology but also contributes to the control of mGluR 1/5 function and LTD. Busch et al. (2023) found that overexpression of CYFIP1, the gene encoding cytoplasmic FMR1, enhanced the localization of NRXN1 at climbing fiber synapse input sites on Purkinje cell primary dendrites. This enhanced localization might reflect the effect of CYFIP1 overexpression on NRXN1 positioning or stability at this site, which affects synaptic signal transmission. Interestingly, *Cyfip1*, the gene encoding cytoplasmic *FMR1*, has been identified as an ASD candidate gene for several years. In addition, the CYFIP1 protein acts as a binding partner for FMRP in the regulation of translation initiation (Busch et al., 2023). CYFIP1 interacts with FMRP to form an inhibitory complex that regulates long-term synaptic plasticity (Aishworiya et al., 2023).

#### 3.3.3 Potential pharmacological targets

The interactions between regulatory mechanisms of NLGNs, FMRP, and NRXNs suggest molecular mechanisms underlying FXS and other neurological disorders, offering potential therapeutic targets for future drug development.

### 3.4 Leucine-rich repeat proteins

#### 3.4.1 Characteristics and function

Leucine-rich repeat transmembrane proteins belong to the synaptic CAMs family and are exclusively expressed in the vertebrate brain (Ko, 2012; Roppongi et al., 2017). All four LRRTM family members regulate the structure, transmission, and plasticity of excitatory synapses in the hippocampus (Ko et al., 2009, 2011; Soler-Llavina et al., 2013; Bhouri et al., 2018; Dhume et al., 2022). LRRTMs likely exert their regulatory effects by binding to presynaptic NRXNs and postsynaptic PSD-95 PDZ proteins (de Wit et al., 2009; Siddiqui et al., 2010; Soler-Llavina et al., 2011; de Arce et al., 2023; Khoja et al., 2023). LRRTM1 is intracellular, whereas LRRTM2 and LRRTM4 are membrane-bound proteins. Further research indicated that LRRTM2 primarily localizes to the postsynaptic membranes of excitatory synapses, and it is more effective than other LRRTMs in inducing presynaptic differentiation (de Wit et al., 2009; Linhoff et al., 2009). Interestingly, LRRTM1 and LRRTM2 cooperate with NLGN1 and NLGN3, whereas LRRTM3 and LRRTM4 bind to NRXNs and together play a crucial role in maintaining normal excitatory synaptic levels through activity-dependent mechanisms (Kim et al., 2022).

#### 3.4.2 Possible implication of LRRTMs in FXS or FMRP pathologies

Parvin et al. (2019) first discovered that LRRTM2 complexes induce the simultaneous accumulation of FMRP and Munc18-1 (a product of the *Stxbp1* gene) in axonal presynapses of cultured mouse cortical neurons. Munc18-1 is an active-zone synaptic vesicle fusion protein, one of the target proteins regulated by FMRP through local translation at presynapses. In the early stages of synaptic development in *Fmr1* KO mice, excessive accumulation of Munc18-1 at presynapses, induced by LRRTM2, may play a crucial role in impairing presynaptic function in FXS (Parvin et al., 2019). Recent research has shown that metformin reduces this exaggerated synaptic release and Munc18-1 accumulation in the presynaptic terminals of neurons in *Fmr1* KO mice (Takeda et al., 2023) and suggests the value of research into the association between LRRTM2 and FXS. Metformin, a medication frequently prescribed to treat type 2 diabetes, has been demonstrated to suppress excessive protein synthesis by inhibiting the mTOR (mammalian target of rapamycin) and ERK pathways. In addition, it has been shown to alleviate core deficiencies in *Fmr1* KO mice, including aberrant spine morphology, exaggerated LTD, and increased repetitive behaviors (Gantois et al., 2017, 2019). Thus, metformin may serve as a potential therapeutic option for FXS.

Using an LRRTM3-deficient mouse model, Kim et al. showed that LRRTM3 may be a key factor in activity-dependent synchronization of excitatory synaptic connections within the medial entorhinal cortex (MEC)-dentate gyrus (DG)-hippocampal CA3 neural circuit by shaping target-specific structures and functional characteristics of specific hippocampal circuits (Kim et al., 2022). Sousa et al. (2010) reported a close relationship between *LRRN3* and *LRRTM3*, which are both rich in neuronal leucine and associated with susceptibility to ASD. *LRRN3* is localized within the genomic region most commonly duplicated in ASD (Clarke and Eapen, 2014). Similarly, research has indicated an association between repetitive LRRTM4 exon endings and features of autism and ASDs (Clarke and Eapen, 2021; Ji et al., 2021), based on single-nucleus RNA sequencing data from postmortem tissue samples of prefrontal and anterior cingulate cortices of patients with ASD and controls, suggesting LRRTM4 as a potential pathogenic genomic target in ASD. This research defines a new approach to studying gene modules involved in the pathogenesis of ASD. Finally, although there is currently no documented biological association between LRRTM3, LRRTM4, and FMRP, research suggests that members of the LRRTM family play a crucial role in synaptic development in ASD and the establishment of neurological circuits (Ji et al., 2021), deepening our understanding of neurodevelopmental disorders.

## 4 Conclusion

In summary, this review outlines specific pathological and biological characteristics that connect synaptic CAMs to FXS. Synaptic CAMs play crucial regulatory roles in synapse formation, differentiation, stability, and plasticity, thereby affecting information processing in neural circuits and cognitive function. Functional compromise of these molecules may result in cognitive impairment. Herein, we highlighted collaborative interactions among different synaptic adhesion protein families that support connections between presynaptic and postsynaptic neurons, thereby affecting the transmission of neural signals and the stability of neural networks. By modulating collaborative interactions among synaptic CAMs and their interactions with FMRP, symptoms of neurological diseases may be reversed (Stan et al., 2010; Bukalo and Dityatev, 2012; Taylor et al., 2020). However, research on specific associations between synaptic CAMs and FXS is limited, despite their biological importance. Further studies, incorporating advanced neurobiological technologies, are needed to explore the mechanisms of action of synaptic CAMs in FXS and other neurodevelopmental diseases, gain a deeper understanding of the pathogenesis of neurodevelopmental disorders, and identify potential drug targets.

## Author contributions

S-YB: Visualization, Writing – original draft, Writing – review & editing, Conceptualization, Investigation, Methodology. D-YZ: Visualization, Writing – original draft, Writing – review & editing, Conceptualization, Investigation, Methodology. MO: Visualization, Writing – original draft, Writing – review & editing, Conceptualization, Investigation, Methodology. YZ: Funding acquisition, Resources, Supervision, Visualization, Writing – review & editing, Conceptualization. WT: Funding acquisition, Supervision, Visualization, Writing – review & editing, Conceptualization, Resources. LX: Funding acquisition, Resources, Supervision, Visualization, Writing – review & editing, Conceptualization.
